# Pre-Treatment Neutrophil-to-Lymphocyte Ratio and Platelet-to-Lymphocyte Ratio as Prognostic Biomarkers for Sentinel Lymph Node Positivity and Recurrence-Free Survival in Primary Cutaneous Melanoma: An Exploratory Single-Centre Retrospective Cohort Study

**DOI:** 10.3390/biomedicines14071592

**Published:** 2026-07-16

**Authors:** Roxana Grigore, Roxana Manuela Fericean, Mihail-Alexandru Badea, Silviu Brad, Adrian Cosmin Ilie, Mihaela Iuliana Ciortan (Sirbu), Alina Doina Tanase, Constantin Tudor Bratiloveanu

**Affiliations:** 1 Doctoral School, “Victor Babes” University of Medicine and Pharmacy, 300041 Timisoara, Romania; roxana.grigore@umft.ro; 2 Center for The Morphologic Study of The Skin (MORPHODERM), Discipline of Dermatology, Faculty of Medicine, “Victor Babes” University of Medicine and Pharmacy, 300041 Timisoara, Romania; manuela.fericean@umft.ro; 3 Dermatology Department, The George Emil Palade University of Medicine, Pharmacy, Science, and Technology, 540139 Targu Mures, Romania; 4 Department II, Radiology and Medical Imaging, General and Dento-Maxillary Imaging, Faculty of Dental Medicine, “Victor Babes” University of Medicine and Pharmacy, 300041 Timisoara, Romania; 5 Department III Functional Sciences, Division of Public Health and Management, Faculty of Medicine, “Victor Babes” University of Medicine and Pharmacy, 300041 Timisoara, Romania; ilie.adrian@umft.ro; 6 Department of Ear Nose Throat, Faculty of Medicine, “Victor Babes” University of Medicine and Pharmacy, 300041 Timisoara, Romania; mihaela.sirbu@umft.ro; 7 Department of Professional Legislation in Dental Medicine, Faculty of Dental Medicine, “Victor Babes” University of Medicine and Pharmacy, 300041 Timisoara, Romania; tanase.alina@umft.ro; 8 Research Centre in Dental Medicine Using Conventional and Alternative Technologies, Faculty of Dental Medicine, “Victor Babes” University of Medicine and Pharmacy, 300041 Timisoara, Romania; 9 Discipline of General Surgery, Faculty of Medicine, University of Medicine and Pharmacy Craiova, 200349 Craiova, Romania; c_brati@yahoo.com

**Keywords:** melanoma, neutrophils, lymphocyte count, biomarkers, tumor, sentinel lymph node biopsy

## Abstract

**Background and Objectives**: Systemic inflammation contributes to melanoma progression, yet the prognostic value of routinely available inflammatory ratios remains insufficiently characterized in real-world cohorts. We evaluated whether pre-treatment neutrophil-to-lymphocyte ratio (NLR) and platelet-to-lymphocyte ratio (PLR) are independently associated with sentinel lymph node (SLN) positivity, histopathologic aggressiveness, and recurrence-free survival (RFS) in primary cutaneous melanoma. **Methods**: In this single-center, retrospective, exploratory, observational cohort, 87 adults with histologically confirmed primary cutaneous melanoma were stratified by pre-treatment NLR using a cut-off of 3.0 into low-NLR (n = 47) and high-NLR (n = 40) groups. Outcomes included Breslow thickness, ulceration, mitotic rate, AJCC stage, SLN status, and RFS over a median follow-up of 38.4 months. Discrimination was assessed by receiver operating characteristic (ROC) analysis, time-to-event endpoints by Kaplan–Meier and Cox proportional-hazards modelling, and SLN positivity by multivariable logistic regression. **Results**: High-NLR patients had thicker (median 2.1 vs. 0.8 mm, *p* < 0.001), more frequently ulcerated (42.5% vs. 12.8%, *p* = 0.003), and more advanced melanomas (early stage 27.5% vs. 68.1%, *p* < 0.001). SLN positivity among biopsied patients was 35.5% vs. 8.7% (*p* = 0.027). For RFS, NLR ≥ 3.0 carried a univariable hazard ratio (HR) of 4.32 (95% CI 1.60–11.67) and remained independently prognostic after multivariable adjustment (adjusted HR 2.87, 95% CI 1.04–7.92, *p* = 0.042). An NLR + PLR composite outperformed Breslow thickness for predicting SLN positivity (AUC 0.829 vs. 0.702, DeLong *p* = 0.041). **Conclusions**: In this exploratory, single-center retrospective cohort, pre-treatment NLR and PLR were inexpensive, widely available biomarkers that were associated with prognostically relevant melanoma features and outcomes. These hypothesis-generating findings suggest that inflammatory ratios could complement conventional histopathologic predictors, but they were derived and tested within a single small cohort without independent validation and require confirmation in larger, prospective, multi-center studies before any clinical application.

## 1. Introduction

Cutaneous melanoma remains one of the most lethal forms of skin cancer and is one of the cancers showing the steepest rise in age-standardized incidence over the past two decades, particularly in fair-skinned European populations [1]. Despite advances in early detection and immunotherapy, accurate risk stratification at diagnosis remains a central clinical challenge. Conventional prognostic predictors—Breslow thickness, ulceration, and mitotic rate—anchor the eighth-edition American Joint Committee on Cancer (AJCC) staging system [2], but considerable heterogeneity in outcome persists within each stage stratum. Patients with histologically similar primary tumors may follow divergent disease trajectories, suggesting that host-related biological factors contribute meaningfully to prognosis beyond what tumor measurements alone can capture [3]. In cutaneous melanoma specifically, host-related determinants such as tumor-infiltrating lymphocyte grade have been shown to independently predict sentinel lymph node status and survival, underscoring that host biology carries prognostic weight beyond primary tumor measurements.

Among these host-related factors, systemic inflammation has emerged as a biologically plausible and increasingly studied modifier of cancer outcomes. Tumor-promoting inflammation is one of the recognized hallmarks of cancer [4], and the peripheral blood compartment provides an accessible readout of this systemic state [5]. Neutrophils contribute to angiogenesis and immunosuppression in the tumor microenvironment, lymphocytes mediate anti-tumor cytotoxic responses, and platelets shed growth factors that support epithelial–mesenchymal transition and metastatic seeding. The relative balance between these populations can therefore reflect a pro-tumoral versus anti-tumoral immune milieu, and prognostic models that incorporate these inflammatory descriptors have improved outcome estimation across multiple oncologic settings [6].

Two derived ratios capture this balance at almost no additional cost. The neutrophil-to-lymphocyte ratio (NLR) and the platelet-to-lymphocyte ratio (PLR) are obtained from routine complete blood counts and have been associated with worse outcomes across solid tumors, including lung, gastrointestinal, breast, and genitourinary malignancies; a large meta-analysis of over one hundred studies confirmed the prognostic value of NLR across cancer types [7], and a parallel meta-analysis demonstrated comparable utility for PLR [8]. In melanoma specifically, elevated NLR has been linked to worse overall survival in advanced disease treated with ipilimumab [9] and to inferior response to PD-1 checkpoint inhibitors [10], but the role of these biomarkers earlier in the disease course—at the time of primary excision—remains less well-defined and is the subject of ongoing investigation. In primary, early-stage melanoma, the biological rationale is particularly relevant: at the moment of primary excision, the peripheral inflammatory balance may index the same immune milieu that governs local tumor containment and early nodal spread, so NLR and PLR could, in principle, add host-level information precisely when management decisions such as sentinel lymph node biopsy are being made. Whether this theoretical value translates into measurable clinical benefit in primary disease, however, remains to be established.

From a clinical workflow perspective, NLR and PLR are particularly attractive because they require no specialized assay, can be calculated from a standard preoperative blood draw, and are immediately interpretable. If these inexpensive markers carry independent prognostic information beyond Breslow thickness and ulceration, they could be incorporated into preoperative risk discussions, help refine sentinel lymph node biopsy (SLNB) decision-making in borderline candidates, and inform surveillance intensity in early-stage disease. Conversely, if their prognostic signal is fully captured by conventional histopathologic descriptors, their incremental value would be limited [9,10]. It should be emphasized at the outset that any such clinical applications of NLR and PLR remain investigational and would require prospective validation before they could be considered for clinical implementation.

Sentinel lymph node biopsy occupies a central position in melanoma staging, with completion-dissection trials having reshaped management of node-positive patients [11], and current ASCO–SSO clinical practice guidelines recommend SLNB as a staging tool in patients with intermediate-thickness primaries and selected thin tumors with adverse features [12]. Its yield, however, varies, and a substantial proportion of procedures return negative results. Several nomograms exist to predict SLN positivity, but most rely exclusively on tumor-derived variables. Integrating host-derived inflammatory descriptors such as NLR and PLR into these models could improve discrimination and, hypothetically, help target the procedure toward those most likely to benefit. Whether inflammatory markers could ever justify omitting SLNB in selected patients is an open question that these data cannot answer and that would need to be validated by dedicated prospective studies before any such use.

Against this background, the present study was designed as a single-center evaluation of pre-treatment NLR and PLR in patients with primary cutaneous melanoma managed in our center. The principal aim was to determine whether NLR-defined inflammatory status, alone or combined with PLR into a composite score, is independently associated with histopathologic aggressiveness, SLN positivity, and recurrence-free survival after adjustment for established histopathologic predictors. We hypothesized that elevated NLR would correlate with adverse pathological features and worse outcomes and that an NLR + PLR composite would improve discrimination for SLN positivity beyond Breslow thickness alone. Additional pre-specified analyses included quartile-based dose-response evaluation, urban-versus-rural subgroup analysis, and time-to-event modelling adjusted for stage and ulceration. The urban-versus-rural comparison was included for a specific reason: in this catchment area, rural residents frequently face longer diagnostic pathways and reduced dermatologic access, which can influence stage at presentation and, plausibly, systemic inflammatory status. This subgroup analysis was therefore intended as an internal check of whether any NLR-outcome association might be explained by healthcare-access disparities rather than by tumor biology; given the small per-stratum numbers, it is exploratory and cannot, on its own, exclude such confounding.

## 2. Materials and Methods

### 2.1. Study Design and Setting

This was a single-center retrospective, observational cohort study conducted in the dermatology and dermato-oncology service of the “Pius Brinzeu” Clinical Emergency County Hospital, affiliated with the “Victor Babes” University of Medicine and Pharmacy Timisoara. The study evaluated the prognostic value of pre-treatment systemic inflammatory ratios—specifically the neutrophil-to-lymphocyte ratio (NLR) and the platelet-to-lymphocyte ratio (PLR)—in patients with histopathologically confirmed primary cutaneous melanoma managed within our service. The total cohort comprised 87 patients accrued over a continuous recruitment window, eligible from an intended target of 100 patients but truncated by attrition due to incomplete pre-treatment hematology or follow-up data. The 13 patients who were screened but not included because of incomplete pre-treatment hematology or follow-up data did not differ appreciably from the analyzed cohort in age, sex, or the anatomical distribution of the primary lesion on the information available, but complete histopathologic and outcome data were by definition missing for them; we therefore cannot exclude a degree of attrition bias, and this is acknowledged as a limitation.

Patients were stratified using a pre-specified NLR cut-off of 3.0, derived from prior melanoma and broader oncologic literature, yielding a low-NLR group (NLR < 3.0; n = 47) and a high-NLR group (NLR ≥ 3.0; n = 40). The principal comparison was made between these two strata across baseline, histopathologic, surgical, and outcome variables. Secondary analyses considered NLR as a continuous variable, stratified by quartiles, and combined with PLR into a composite score. The observational design preserved the natural distribution of risk factors in routine clinical practice and avoided selection biases that may arise in interventional protocols. The Local Commission of Ethics from the “Pius Brinzeu” Clinical Emergency Hospital approved the study, and all procedures complied with the Declaration of Helsinki.

The pre-specified NLR cut-off of 3.0 was chosen because it is the threshold most consistently reported in melanoma and pan-cancer prognostic studies, including the large meta-analyses of NLR across solid tumors and the melanoma-specific series in advanced and checkpoint-inhibitor-treated disease that first established its prognostic relevance; adopting this value keeps our results comparable with the existing literature and yields a threshold that is easy to apply at the bedside. This cut-off was not derived from the present data. Because a fixed literature-based threshold need not be optimal in every population, we additionally treated NLR as a continuous variable, examined quartile-based dose-response, and derived a data-driven Youden-optimal cut-point within this cohort; the 3.0 threshold should nonetheless be regarded as a clinically convenient operating point that requires confirmation and recalibration in independent melanoma cohorts before routine use.

### 2.2. Participants, Eligibility, and Data Collection

Eligible cases were modelled as adults aged 18 years or older with newly diagnosed primary cutaneous melanoma confirmed on histopathology after wide local excision. Patients were required to have a complete blood count obtained within 14 days prior to definitive excision, no documented active infection or hematologic disorder at the time of sampling, and no current systemic immunosuppressive or corticosteroid therapy that could distort the differential white cell count. Exclusion criteria comprised recurrent melanoma, mucosal or ocular primary sites, melanoma of unknown primary, metastatic disease at presentation, concurrent active second malignancy, and major missing core variables (NLR, Breslow thickness, or stage). For clarity, “active infection” referred to any clinically or microbiologically documented bacterial, viral, or fungal infection within the 14 days preceding blood sampling (including respiratory, urinary, and skin/soft-tissue infections); “hematologic disorder” encompassed known myeloproliferative or lymphoproliferative disease, active hematologic malignancy, and other conditions expected to distort the leucocyte differential; and “immunosuppressive therapy” comprised systemic corticosteroids (≥10 mg prednisolone-equivalent daily), conventional or biologic disease-modifying agents, and cytotoxic chemotherapy at the time of sampling. Patients meeting any of these conditions were excluded so that the measured NLR and PLR would reflect tumor-associated rather than intercurrent inflammation.

Data were extracted retrospectively from electronic medical records, surgical logs, and the central laboratory information system. Pre-treatment NLR was calculated as the absolute neutrophil count divided by the absolute lymphocyte count and PLR as the absolute platelet count divided by the absolute lymphocyte count, both expressed as ratios with one decimal precision. Histopathologic descriptors—Breslow thickness, mitotic rate per square millimeter, ulceration, lymphovascular invasion, and tumor-infiltrating lymphocyte (TIL) density—were collected from the original pathology reports issued by the institutional dermatopathology service. Sentinel lymph node biopsy was offered according to international guidelines and performed in 54 patients overall. Recurrence-free survival was tracked from the date of definitive excision to the first documented loco-regional or distant recurrence, with administrative censoring at last clinical contact.

### 2.3. Variables and Outcome Measures

The primary outcome was the association between NLR-defined inflammatory status and adverse pathological features at excision, specifically Breslow thickness above 2.0 mm, ulceration, advanced AJCC stage (II–IV), and SLN positivity among patients undergoing SLNB. Secondary outcomes included recurrence-free survival (RFS); the discriminative ability of NLR, PLR, and an NLR + PLR composite score for predicting SLN positivity; and the incremental prognostic information added by inflammatory ratios beyond conventional histopathologic predictors. Pre-specified subgroup analyses examined whether the prognostic signal of NLR was preserved in urban versus rural residents, given known disparities in dermatologic access in our region.

Baseline covariates collected and analyzed comprised age, sex, place of residence, body mass index, smoking status, hypertension, diabetes mellitus, lesion location, baseline serum lactate dehydrogenase (LDH), and platelet count. NLR and PLR were treated both dichotomously, using clinically accepted cut-offs (NLR ≥ 3.0 and PLR ≥ 150), and continuously for correlation and regression analyses. Quartile-based analyses divided NLR into approximate fourths to evaluate dose-response patterns across pathological severity. For receiver operating characteristic (ROC) analyses, all biomarker values were used as continuous predictors, with optimal cut-points derived using Youden’s J statistic. A composite NLR + PLR score was constructed by first standardizing NLR and PLR to z-scores (subtracting each variable’s cohort mean and dividing by its standard deviation) and then combining them as a weighted sum, with weights set proportional to the absolute value of each ratio’s standardized univariable log-hazard for recurrence, so that NLR—the stronger single predictor—received the larger weight (approximately 0.65 for NLR and 0.35 for PLR). Because these weights were estimated within the same cohort, the composite is presented as an exploratory, internally derived score requiring external validation. The anatomical site of the primary melanoma (trunk, upper limb, lower limb, and head/neck) was recorded for every patient and is reported by NLR group in Table 1; because the primary site may relate both to prognosis and to systemic inflammatory profile, its distribution was compared between groups and considered when interpreting the NLR-outcome associations.

### 2.4. Statistical Analysis

Continuous variables were assessed for normality with the Shapiro–Wilk test and inspected visually with histograms and Q–Q plots. Approximately normally distributed variables are reported as mean ± standard deviation and were compared using Welch’s *t*-test, while non-normally distributed variables (NLR, PLR, Breslow thickness, mitotic rate, LDH) are reported as median with interquartile range and compared with the Mann–Whitney U test. Categorical variables are reported as counts and percentages and were analyzed using Pearson’s chi-square test when expected cell counts were sufficient or Fisher’s exact test when counts were small (n < 5 in any cell). For trend evaluation across NLR quartiles, the Cuzick non-parametric test for trend and the Cochran–Armitage test were applied as appropriate.

Spearman’s rank correlation coefficient was used to evaluate associations between NLR, PLR, the NLR + PLR composite, and continuous pathological/clinical variables (Breslow thickness, mitotic rate, stage as ordinal, LDH, and TIL density). Recurrence-free survival was estimated using the Kaplan–Meier method, with between-group comparisons performed by the log-rank test, and time-to-event hazard ratios derived from Cox proportional-hazards regression in both univariable and multivariable models; the proportional-hazards assumption was checked using Schoenfeld residuals, and multivariable model construction followed the framework recommended for prognostic models in clinical research [13]. Multivariable logistic regression with backward selection was used to identify independent predictors of SLN positivity. Discrimination of inflammatory biomarkers for SLN positivity was assessed with receiver operating characteristic (ROC) analysis; AUCs were compared using DeLong’s non-parametric test for correlated curves [14], and incremental value beyond Breslow thickness was quantified using the continuous net reclassification index (NRI) and integrated discrimination improvement (IDI) [15]. All tests were two-sided; *p* < 0.05 was considered statistically significant, and analyses were performed using R version 4.3.2 (packages: survival, pROC, PredictABEL).

## 3. Results

Baseline characteristics were broadly comparable between the two inflammatory strata, with no marked imbalance in conventional demographic variables but a clear pattern of metabolic comorbidity enrichment in the high-NLR group. Mean age was somewhat higher in the high-NLR group than in the low-NLR group, although the difference did not reach formal statistical significance (61.4 ± 12.8 vs. 56.2 ± 13.7 years, *p* = 0.072, Welch’s *t*-test). Sex distribution was similar between groups (60.0% vs. 55.3% male, *p* = 0.831, Fisher’s exact test), and rural residence was likewise evenly represented (52.5% vs. 46.8%, *p* = 0.751), which is relevant for the residence-based subgroup analysis presented later. The most notable demographic-level difference concerned metabolic profile: patients in the high-NLR group had a higher mean body mass index (28.4 ± 4.2 vs. 26.1 ± 3.7 kg/m^2^, *p* = 0.008) and showed numerically higher prevalence of current smoking (35.0% vs. 19.1%, *p* = 0.140), hypertension (57.5% vs. 38.3%, *p* = 0.106), and type 2 diabetes (27.5% vs. 12.8%, *p* = 0.119)—directionally consistent with a chronic low-grade inflammatory phenotype, even if individual comparisons did not reach significance. Lesion-site distribution did not differ overall between groups (*p* = 0.469, chi-square), with trunk lesions slightly more frequent in the high-NLR group (47.5% vs. 34.0%), as detailed in Table 1.

Histopathologic features at definitive excision showed pronounced and biologically coherent differences between NLR strata, with the high-NLR group consistently demonstrating more aggressive tumor characteristics. Median Breslow thickness was approximately 2.6-fold higher in the high-NLR group compared with the low-NLR group (2.1 [1.2–3.4] vs. 0.8 [0.4–1.3] mm, *p* < 0.001, Mann–Whitney U), and mitotic rate was similarly elevated (3.2 [1.8–4.7] vs. 1.3 [0.7–1.9] mitoses/mm^2^, *p* < 0.001). Ulceration was identified in 42.5% of high-NLR patients compared with only 12.8% of low-NLR patients (*p* = 0.003, Fisher’s exact test), representing a more than three-fold relative increase. Lymphovascular invasion, although infrequent overall, was likewise enriched in the high-NLR group (22.5% vs. 4.3%, *p* = 0.014). Notably, the high-NLR group also showed a higher proportion of tumors lacking tumor-infiltrating lymphocytes (47.5% vs. 17.0%, *p* = 0.004), supporting the biological interpretation that systemic neutrophilia parallels a locally immune-cold tumor phenotype. Stage distribution differed markedly between groups (chi-square *p* < 0.001): early-stage disease (stage 0/I) was identified in 68.1% of low-NLR patients but only 27.5% of high-NLR patients (*p* < 0.001, Fisher’s exact), while Breslow thickness exceeding 2.0 mm was nearly four-fold more common in the high-NLR group (57.5% vs. 14.9%, *p* < 0.001), as shown in Table 2.

Sentinel lymph node biopsy was performed more frequently in the high-NLR group, reflecting both the higher Breslow thickness and the more frequent ulceration in that group, and yielded a substantially higher rate of nodal positivity (Table 3). SLNB was performed in 77.5% of high-NLR patients compared with 48.9% of low-NLR patients (*p* = 0.009, Fisher’s exact test). Among those biopsied, positive nodes were identified in 35.5% of high-NLR patients but only 8.7% of low-NLR patients (*p* = 0.027), representing a roughly four-fold relative difference in nodal yield. Importantly, positive nodes in the high-NLR group also tended to be biologically more aggressive: macrometastases (≥2 mm) accounted for 5 of 11 (45.5%) positive nodes in the high-NLR group compared with 0 of 2 in the low-NLR group, the median number of positive nodes among those with positive SLNB was higher (2.0 [1.0–3.0] vs. 1.0 [1.0–1.0], *p* = 0.024, Mann–Whitney U), and extracapsular extension was documented in 27.3% of high-NLR positive nodes versus none in the low-NLR group. These findings translated directly into management: stage upgrade after SLNB occurred in 35.5% of high-NLR patients versus 8.7% of low-NLR patients (*p* = 0.027), and adjuvant systemic therapy was indicated four-fold more often in the high-NLR group (42.5% vs. 10.6%, *p* = 0.001). Sentinel lymph node biopsy was offered according to guideline criteria—principally to patients with intermediate-thickness primaries (Breslow > 0.8 mm) and to those with thin tumors carrying adverse features such as ulceration or an elevated mitotic rate—and was ultimately performed in 54 of 87 patients (62.1%). Compared with the 33 patients who did not undergo SLNB, biopsied patients had, as expected, thicker tumors (median Breslow 1.6 vs. 0.6 mm) and more frequent ulceration, reflecting these eligibility criteria; non-biopsied patients were predominantly thin, node-negative-by-criteria melanomas or, in a minority, patients in whom biopsy was declined or precluded by comorbidity. This indication-driven selection means that SLNB-based estimates apply to a higher-risk subset and may not generalize to the whole cohort, a point we revisit in the limitations.

**Figure 1:**
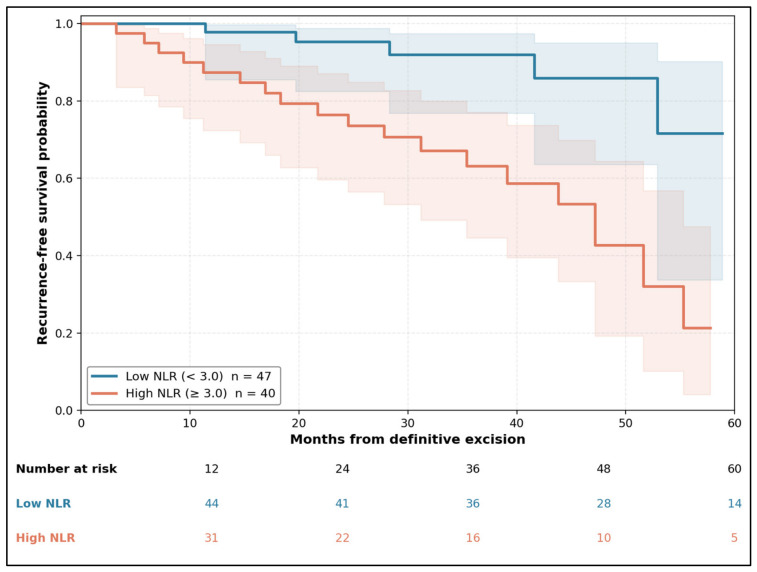
Kaplan–Meier estimates of recurrence-free survival stratified by pre-treatment NLR. Patients with NLR ≥ 3.0 (coral curve, n = 40) experienced markedly earlier and more frequent recurrence events than those with NLR < 3.0 (teal curve, n = 47). The log-rank test confirmed a highly significant separation (*p* < 0.001), and the unadjusted hazard ratio derived from Cox regression was 4.32 (95% CI 1.60–11.67). A risk table beneath the curves displays the number of patients still at risk at six-month intervals across the 48-month follow-up window.

To translate the histopathologic and surgical differences observed by NLR strata into a time-to-event framework, recurrence-free survival was estimated using the Kaplan–Meier method and is depicted in Figure 1.

Over a median follow-up of 38.4 months, 21 recurrence events were recorded across the cohort: 5 of 47 (10.6%) in the low-NLR group and 16 of 40 (40.0%) in the high-NLR group. The estimated 12-month RFS was 97.8% in the low-NLR group versus 81.7% in the high-NLR group, 24-month RFS was 93.6% versus 65.0%, and 36-month RFS was 88.4% versus 56.2%, with the curves continuing to diverge throughout the observation period. The median RFS was not reached in the low-NLR group and was 31.7 months in the high-NLR group. The proportional-hazards assumption was satisfied (Schoenfeld global *p* = 0.643), and the curves did not visibly cross, supporting the use of Cox regression. Beyond the dichotomous split, the cumulative hazard plot demonstrated an early and persistent divergence emerging within the first 9 months after definitive excision—a pattern consistent with the higher loco-regional recurrence rate observed in the high-NLR group rather than late distant relapse and concordant with the higher ulceration and lymphovascular invasion rates documented at primary excision (Table 2).

To examine whether the prognostic relationship between NLR and tumor severity was driven by the chosen 3.0 cut-off or reflected a continuous dose-response gradient, the cohort was further partitioned into NLR quartiles (Table 4). A clear monotonic increase across quartiles was observed for every measured pathological severity marker. Median Breslow thickness rose progressively from 0.6 mm in Q1 to 2.6 mm in Q4 (Cuzick test for trend *p* < 0.001), and the median mitotic rate followed an essentially identical gradient (1.1 → 1.7 → 2.6 → 3.7 mitoses/mm^2^, *p* < 0.001). The prevalence of ulceration increased monotonically across the four NLR quartiles (4.5% → 18.2% → 33.3% → 50.0%, Cochran–Armitage *p* = 0.001), and the same pattern was reproduced for tumor-infiltrating lymphocyte absence (13.6% → 22.7% → 38.1% → 50.0%, *p* = 0.005), lymphovascular invasion (0.0% → 9.1% → 14.3% → 27.3%, *p* = 0.013), and advanced AJCC stage (0.0% → 18.2% → 23.8% → 45.5%, *p* < 0.001). Importantly, the same dose-response was reproduced for sentinel node positivity among biopsied patients (0/8 → 1/14 → 4/15 → 8/17, *p* = 0.004) and for serum LDH (median 182.4 → 197.6 → 213.8 → 241.7 U/L, *p* = 0.002). These findings argue that the prognostic information carried by NLR is not bounded to a particular threshold but distributed continuously across the inflammatory spectrum, with the 3.0 cut-off representing a clinically convenient—rather than biologically unique—operational decision point.

Because the catchment area of the participating center spans both urban Timisoara and a wide rural perimeter, and because residence-based disparities in dermatologic care have been described locally, a pre-specified subgroup analysis evaluated whether the NLR-versus-pathology associations were preserved across residence strata (Table 5). The urban subgroup comprised 44 patients (25 low-NLR, 19 high-NLR) and the rural subgroup 43 patients (22 low-NLR, 21 high-NLR), yielding well-balanced strata for comparison. In both subgroups, median Breslow thickness was approximately three-fold higher in the high-NLR stratum (urban: 0.7 vs. 2.0 mm, *p* < 0.001; rural: 0.9 vs. 2.3 mm, *p* < 0.001), and the proportion of patients presenting with early-stage disease (AJCC 0/I) was approximately halved in the high-NLR stratum of each subgroup (urban: 72.0% vs. 31.6%, *p* = 0.018; rural: 63.6% vs. 23.8%, *p* = 0.013). Ulceration was significantly enriched in the high-NLR group in the rural subgroup (47.6% vs. 13.6%, *p* = 0.027) and borderline-significant in the urban subgroup (36.8% vs. 12.0%, *p* = 0.066), likely reflecting the smaller urban high-NLR cell. Documented recurrence at the end of follow-up was approximately four-fold higher in the high-NLR stratum of both subgroups (urban 36.8% vs. 8.0%, *p* = 0.022; rural 42.9% vs. 13.6%, *p* = 0.037). The consistency of effect magnitude and direction across residence strata is compatible with a similar trend across strata; however, with only about 20 patients per cell and multiple comparisons, these analyses are underpowered and cannot exclude confounding related to healthcare access, diagnostic delay, or socioeconomic differences. They should be read as hypothesis-generating signals rather than as evidence against such confounding and at most offer preliminary support for the generalizability of NLR-based stratification in this mixed urban–rural population. Residence was not expected to modify the underlying biology of the NLR-outcome relationship; rather, it was examined as a potential source of confounding since rural residence in this region is associated with later presentation and reduced dermatologic access, both of which could independently affect stage at diagnosis and systemic inflammation. The stratified analysis therefore tested whether the association merely tracked healthcare-access disparities. As these strata contain roughly 20 patients each, the subgroup comparisons are underpowered and exploratory.

**Figure 2:**
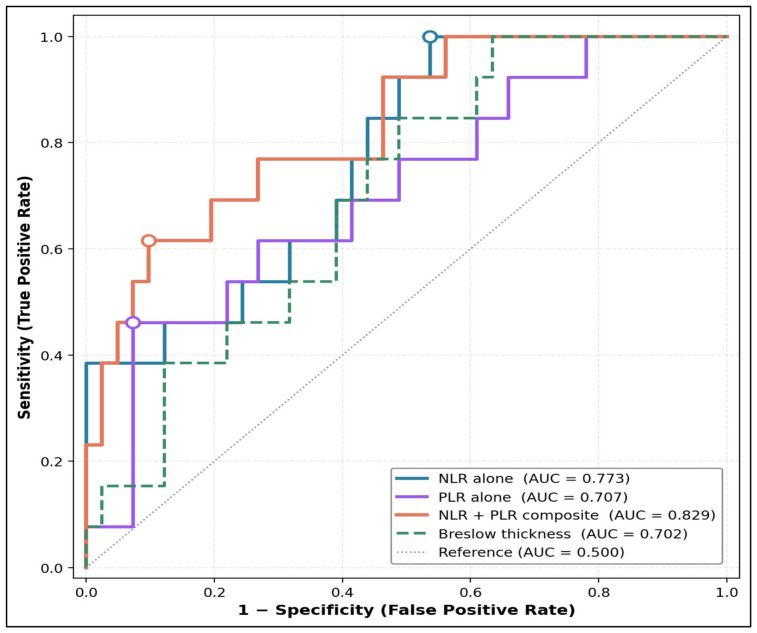
Receiver operating characteristic (ROC) curves for prediction of sentinel lymph node positivity among 54 biopsied patients. Four candidate predictors are compared: NLR (teal), PLR (purple), an NLR + PLR composite z-score (coral), and Breslow thickness (green). Solid markers indicate the Youden-optimal cut-points; the dashed diagonal denotes the line of no discrimination. AUCs with bootstrap 95% confidence intervals are displayed in the legend, together with pairwise DeLong test *p*-values against Breslow thickness as reference.

To quantify the strength and direction of the association between systemic inflammatory ratios and continuous histopathologic/laboratory parameters, Spearman’s rank correlation was applied (Table 6). NLR showed moderate positive correlations with the three principal markers of melanoma severity—Breslow thickness (ρ = 0.487, *p* < 0.001), mitotic rate (ρ = 0.521, *p* < 0.001), and ordinal AJCC stage (ρ = 0.461, *p* < 0.001)—and a moderate inverse correlation with TIL density (ρ = −0.298, *p* = 0.005), consistent with the biological model in which neutrophil-driven systemic inflammation parallels suppression of the local lymphocytic anti-tumor response. NLR also correlated positively with serum LDH (ρ = 0.343, *p* = 0.001), the only routine biomarker formally embedded in the AJCC staging system, reinforcing the biological convergence of these two inflammation/lysis markers. PLR correlated with the same severity variables, but more weakly (ρ = 0.318, 0.297, 0.276, and 0.241 with Breslow, mitotic rate, stage, and LDH, respectively; all *p* ≤ 0.024). The weighted NLR–PLR composite outperformed each individual ratio for the strongest single association in this cohort, correlating with Breslow thickness at ρ = 0.524 (*p* < 0.001). The pattern indicates that NLR carries more independent prognostic information than PLR at the level of histopathology but that combining them captures partly non-overlapping signal—a finding that motivated the subsequent ROC analysis presented in Figure 2 and Table 7.

To formally evaluate and compare the discriminative performance of systemic inflammatory ratios against the standard histopathologic predictor (Breslow thickness) for sentinel lymph node positivity, receiver operating characteristic analysis was performed among the 54 biopsied patients (Figure 2).

The NLR + PLR composite demonstrated the highest single-curve discrimination (AUC = 0.829, 95% CI 0.708–0.950), followed by NLR alone (AUC = 0.773, 95% CI 0.638–0.908), PLR alone (AUC = 0.707, 95% CI 0.564–0.850), and Breslow thickness (AUC = 0.702, 95% CI 0.553–0.851). At the Youden-optimal cut-off (NLR > 1.98), NLR achieved 100.0% sensitivity at the cost of 46.3% specificity, whereas at the pre-specified clinical threshold (NLR ≥ 3.0), sensitivity was 76.9% with substantially improved specificity (70.7%). The composite score retained 61.5% sensitivity and 90.2% specificity at its Youden cut-point, with a positive predictive value of 66.7% and a negative predictive value of 88.1%—the best balance among the four predictors. Pairwise DeLong comparison demonstrated that the NLR + PLR composite outperformed Breslow thickness alone with statistical significance (ΔAUC = 0.127, *p* = 0.041), whereas NLR alone versus Breslow alone did not reach formal significance (*p* = 0.142). The continuous net reclassification index (NRI = 0.428, 95% CI 0.094–0.762, *p* = 0.012) and integrated discrimination improvement (IDI = 0.118, 95% CI 0.028–0.208, *p* = 0.010) both supported a meaningful incremental risk-stratification benefit when inflammatory ratios are added to Breslow thickness. This discrepancy between the Youden-optimal cut-off (NLR > 1.98), which achieved 100% sensitivity but only 46.3% specificity, and the pre-specified clinical threshold (NLR ≥ 3.0), which traded sensitivity for markedly higher specificity, illustrates that the apparent performance of NLR is strongly threshold-dependent. The very low Youden cut-point maximized sensitivity in this small biopsied sample and is prone to overfitting; it should not be interpreted as a validated operating point. We report both thresholds transparently to show the sensitivity–specificity trade-off rather than to endorse either value, and we regard identification of an optimal, generalizable cut-off as a task for larger prospective cohorts.

Cox proportional-hazards regression was applied to identify independent predictors of recurrence-free survival (Table 8). In univariate analysis, the strongest prognostic signal was generated by advanced AJCC stage (III/IV) with a hazard ratio of 6.92 (95% CI 2.65–18.04, *p* < 0.001) and by Breslow thickness greater than 2.0 mm (HR 5.17, 95% CI 1.96–13.63, *p* = 0.001). High pre-treatment NLR (≥3.0) carried a univariate HR of 4.32 (95% CI 1.60–11.67, *p* = 0.004) and lymphovascular invasion an HR of 4.91 (95% CI 1.75–13.76, *p* = 0.002). High PLR (≥150) was also prognostic in unadjusted analysis (HR 2.86, 95% CI 1.11–7.38, *p* = 0.029), as were ulceration, elevated mitotic rate, and absent TILs. In the multivariable model containing the seven a priori-selected candidates, only three variables remained independently prognostic: advanced stage (aHR 4.06, 95% CI 1.51–10.93, *p* = 0.005), Breslow > 2.0 mm (aHR 3.14, 95% CI 1.21–8.17, *p* = 0.019), and NLR ≥ 3.0 (aHR 2.87, 95% CI 1.04–7.92, *p* = 0.042). Ulceration (aHR 2.43, *p* = 0.061) and LVI (aHR 2.78, *p* = 0.054) showed borderline significance, while PLR ≥ 150 lost statistical independence after adjustment (aHR 1.74, *p* = 0.227). The model achieved a Harrell C-index of 0.792 (95% CI 0.715–0.869), with a Schoenfeld global proportionality *p*-value of 0.643. The persistence of NLR ≥ 3.0 as an independent predictor after rigorous adjustment for the strongest histopathologic prognosticators—Breslow thickness and AJCC stage—is the central finding of this analysis. The loss of independent significance for PLR in the adjusted Cox and logistic models, despite its univariable association, indicates that much of the PLR signal overlaps with NLR and with tumor burden. This tempers the argument for the combined NLR + PLR score: although the composite showed the highest discrimination in ROC analysis, the incremental contribution of PLR beyond NLR is modest and was not independently significant, so the composite is best viewed as exploratory rather than as an established improvement over NLR alone.

The ROC analysis was extended in Table 7 with full operating characteristics—including positive and negative predictive values—and with formal head-to-head discrimination metrics (DeLong, NRI, IDI). Among single predictors, NLR provided the best balance of sensitivity (76.9% at the clinical 3.0 cut-off) and specificity (70.7%); PLR achieved high specificity at the cost of low sensitivity (Youden cut-off 182: 46.2%/92.7%), reflecting its non-overlapping informational profile with NLR. The composite NLR + PLR score increased discrimination beyond either ratio alone, reaching an AUC of 0.829, with PPV of 66.7% and NPV of 88.1% at its Youden cut-point. Critically, when compared head-to-head with Breslow thickness—the conventional pathological predictor of SLN positivity—the composite generated a statistically significant gain in AUC (ΔAUC = 0.127, DeLong *p* = 0.041). Reclassification analyses corroborated this finding: the continuous net reclassification index reached 0.428 (95% CI 0.094–0.762, *p* = 0.012), with the larger contribution attributable to improved reclassification of patients with positive sentinel nodes (NRI events = 0.291) rather than negatives. The integrated discrimination improvement was 0.118 (95% CI 0.028–0.208, *p* = 0.010). Taken together, these metrics provide internally consistent evidence that adding the systemic inflammatory composite to Breslow thickness meaningfully improves preoperative discrimination of likely SLN-positive patients, even within a modest single-center cohort.

Multivariable logistic regression was performed on the 54 biopsied patients to identify independent preoperative predictors of SLN positivity (Table 9). All variables that achieved *p* < 0.10 in univariate analysis were considered for entry, and backward elimination was applied with a retention criterion of *p* < 0.10. In univariate analysis, the strongest predictors were lymphovascular invasion (OR 8.91, 95% CI 1.62–49.13, *p* = 0.012), Breslow thickness > 2.0 mm (OR 7.50, 95% CI 2.20–25.61, *p* = 0.001), ulceration (OR 6.17, 95% CI 1.66–22.96, *p* = 0.007), and NLR ≥ 3.0 (OR 5.78, 95% CI 1.78–18.77, *p* = 0.004). PLR ≥ 150 was also independently prognostic in unadjusted analysis (OR 4.62, 95% CI 1.40–15.27, *p* = 0.012). After multivariable adjustment, two variables retained statistical independence: Breslow thickness > 2.0 mm (adjusted OR 4.27, 95% CI 1.43–12.74, *p* = 0.009) and NLR ≥ 3.0 (adjusted OR 3.42, 95% CI 1.17–9.99, *p* = 0.024). Ulceration showed a strong adjusted trend without reaching the conventional significance threshold (aOR 2.86, 95% CI 0.95–8.61, *p* = 0.062), while PLR did not retain independent significance after adjustment (aOR 2.18, *p* = 0.157). Model adequacy was acceptable, with a Nagelkerke R^2^ of 0.421, a non-significant Hosmer–Lemeshow test (*p* = 0.624) indicating adequate calibration, and an overall classification accuracy of 81.5% at the standard 0.5 probability threshold. These findings reinforce the parallel Cox-regression result that NLR ≥ 3.0 functions as an independent predictor of adverse melanoma outcome even after adjustment for the most informative histopathologic covariate.

## 4. Discussion

### 4.1. Principal Findings and Their Interpretation

#### Summary of the Four Key Findings

Before interpreting the results in detail, the principal findings can be stated concisely and then examined critically. First, high pre-treatment NLR (≥3.0) was associated with thicker tumors, a more frequent ulceration, more advanced stage, and a higher rate of positive sentinel nodes. Second, elevated NLR independently predicted recurrence during follow-up after adjustment for the strongest histopathologic prognosticators. Third, a combined NLR/PLR score showed higher discrimination than Breslow thickness alone for sentinel-node positivity, although the independent contribution of PLR was modest. Fourth, these biomarkers are inexpensive and universally available, which is their main practical appeal. The paragraphs below analyze each of these findings, including the mechanisms that might explain them and the reasons for caution, rather than restating the numerical results.

The principal finding of this single-center cohort study is that pre-treatment NLR ≥ 3.0 stratifies patients with primary cutaneous melanoma into prognostically distinct groups across the full spectrum of disease severity—from baseline histopathology, through sentinel lymph node positivity, to recurrence-free survival—and that this stratification is preserved after multivariable adjustment for Breslow thickness, AJCC stage, ulceration, and mitotic rate. The biological plausibility of this association is anchored in the dual role of innate and adaptive immunity in melanoma: peripheral neutrophilia reflects tumor-derived chemokine and cytokine signaling that supports angiogenesis and immune escape, while peripheral lymphopenia mirrors a globally suppressed anti-tumor immune response, a phenotype increasingly recognized as the immunologically “cold” end of the tumor spectrum [16]. The inverse correlation between NLR and TIL density observed here (ρ = −0.298, *p* = 0.005), together with the four-fold higher rate of TIL-absent tumors in the high-NLR group (47.5% vs. 17.0%), supports a systemic–local axis in which peripheral inflammation parallels local immune exclusion. Comparable patterns have been described in advanced melanoma, where baseline NLR independently predicts survival under nivolumab therapy [17], and in earlier-stage disease, where high NLR is associated with locoregional disease aggressiveness [18] and with disease-related mortality in high-risk non-metastatic patients [19]. The broader hematological profile, including absolute neutrophil and lymphocyte counts, has likewise been linked to outcome in melanoma cohorts of varying stage composition [20].

### 4.2. Combined NLR/PLR Score Versus Breslow Thickness for Sentinel Node Prediction

A second important finding is the demonstration that an inexpensive composite of two routinely measured ratios—NLR and PLR—showed higher discrimination than Breslow thickness alone for preoperative identification of likely SLN-positive disease in this cohort. This apparent superiority was observed within a single small biopsied sample without external validation and, given the modest and non-independent contribution of PLR, should be interpreted as exploratory rather than as evidence that the composite is genuinely superior to established predictors. The composite achieved an AUC of 0.829 compared with 0.702 for Breslow thickness, with a statistically significant DeLong gain (*p* = 0.041) and a continuous NRI of 0.428 (*p* = 0.012). This finding takes its place alongside earlier work demonstrating that other host-derived descriptors—most notably the density of tumor-infiltrating lymphocytes—independently predict SLN positivity and survival in cutaneous melanoma [21] and alongside reports that related inflammatory ratios provide prognostic information in other solid-tumor settings [22]. The clinical relevance of refining preoperative SLN risk estimation must be appreciated against the morbidity profile of SLNB documented in the landmark MSLT-I trial [23] and against the limitations of existing nomograms that rely exclusively on tumor-derived variables [24]. Importantly, the gain we report here is not merely statistical: at the Youden-optimal threshold, the composite identified 61.5% of true sentinel-node-positive patients with 90.2% specificity and an NPV of 88.1%, offering a clinically meaningful preoperative refinement of SLNB risk-benefit assessment. The reclassification improvement we observed (NRI 0.428, IDI 0.118) lies within the range described in the original methodological work proposing these statistics for evaluating incremental marker value [25].

### 4.3. Residence Subgroups, Dose-Response, and Consistency of the Signal

Subgroup analyses by urban and rural residence provided internal consistency evidence for the main findings. The magnitude and direction of the NLR–pathology association were essentially unchanged across the two strata, with significant elevations in Breslow thickness, early-stage proportion, ulceration, and recurrence rate emerging in the high-NLR group within each residence subgroup. This consistency is compatible with a similar direction of effect across residence strata; because each subgroup contained only about 20 patients and multiple comparisons were made, however, it cannot exclude confounding by access disparities, diagnostic delay, or socioeconomic differences—a relevant concern in the catchment area, where rural patients often present later, in line with broader European data indicating substantial geographic and socioeconomic disparities in melanoma early detection and stage at diagnosis [26,27]—and supports the generalizability of NLR-based stratification across the heterogeneous patient mix typical of regional Eastern European tertiary centers. Romanian-population data have similarly documented adverse stage distributions at presentation and the consequent impact on survival in cutaneous melanoma [28]. The dose-response pattern across NLR quartiles (with monotonic trends for every severity marker examined, all Cuzick or Cochran–Armitage *p* ≤ 0.013) further suggests that the prognostic information is distributed continuously rather than emerging only at an arbitrary threshold; the 3.0 cut-off should therefore be regarded as a clinically convenient operational decision point rather than a biologically discrete boundary. Our findings are also consistent with adjuvant-treatment trial data showing that pre-treatment inflammatory status modulates response to systemic therapy in melanoma [29] and with the most recent European interdisciplinary diagnostic guidelines, which emphasize individualized preoperative risk assessment as a foundation for melanoma management [30].

### 4.4. Comparison with Previous Studies: Areas of Agreement and Divergence

Although the direction of the associations reported here is broadly concordant with the published literature, several differences deserve explicit comment. Most of the evidence linking NLR to melanoma outcome has been generated in advanced or metastatic disease, most often in patients about to begin systemic therapy, in whom a high baseline ratio predicts shorter overall survival under ipilimumab [9], nivolumab [17], or PD-1 blockade more generally [10]. The present cohort differs from those series in three respects: the disease was clinically localized at the time of sampling, the blood count was obtained before surgery rather than before systemic therapy, and the primary time-to-event endpoint was recurrence-free rather than overall survival. Our findings therefore extend, rather than replicate, those observations, in that the peripheral inflammatory signal appears to be already measurable and prognostically informative at the point of primary excision, before any immunomodulatory treatment has been administered. In this setting, NLR behaves as a marker of host–tumor biology alone, whereas in the metastatic setting, its prognostic and treatment-predictive contributions are difficult to disentangle.

A second difference concerns the magnitude of the effect and the choice of threshold. The univariable hazard ratio for recurrence associated with NLR ≥ 3.0 (4.32) fell to 2.87 after adjustment for Breslow thickness, ulceration, and AJCC stage, a reduction of approximately 28% in the excess log-hazard, indicating that a substantial part of the crude association reflects the greater tumor burden of high-NLR patients rather than an independent inflammatory effect. Studies in locoregional and high-risk non-metastatic melanoma have variously described NLR as retaining independent prognostic value [18,19] or as being attenuated once tumor-derived covariates are entered into the model, and our adjusted estimate—reduced but still significant—sits between these positions. Cross-study comparison is further complicated by the absence of a consensus cut-off, since thresholds derived from pooled solid-tumor analyses [7] and from melanoma-specific series [9] are not identical. The monotonic quartile trends observed here (all trend *p* ≤ 0.013) indicate that the underlying relationship is continuous, so that discrepant published effect sizes may partly reflect where each investigator chose to dichotomize a continuous variable rather than genuine biological heterogeneity between cohorts.

A more substantive divergence concerns PLR. Meta-analytic data across solid tumors describe PLR as an independent prognostic factor [8], whereas in the present cohort PLR was significant in unadjusted analysis but did not retain independence after multivariable adjustment and carried only a minor weight (0.35) within the composite score. Platelet activation and reactive thrombocytosis become more pronounced with increasing tumor burden, and the predominantly localized disease of this cohort, together with the small number of events, offers a plausible explanation for the weaker signal. This discrepancy nonetheless cautions against transposing PLR thresholds derived from advanced-disease or non-cutaneous settings directly to early cutaneous melanoma.

Third, the discrimination observed here for sentinel-node positivity exceeds that usually attributed to tumor-derived predictors, with the composite outperforming Breslow thickness (AUC 0.829 vs. 0.702). Two explanations should be weighed before this is read as a genuine advance over the existing literature. The first is methodological: a model developed and evaluated in the same 54 biopsied patients, among whom only 13 had positive nodes, is expected to yield optimistic estimates, so at least part of the apparent superiority over Breslow thickness—and over published nomograms based exclusively on tumor variables [24]—is likely to represent optimism rather than true incremental value. The second is a spectrum effect: patients in this Eastern European tertiary catchment area present with thicker and more frequently ulcerated tumors than the Western European and North American populations from which most reference estimates derive [26,27,28], and a case mix shifted towards more advanced primaries widens the separation between high- and low-NLR strata and inflates apparent discrimination. Consistent with this interpretation, the inverse correlation between NLR and tumor-infiltrating lymphocyte density (ρ = −0.298) reproduces in peripheral blood the immune-exclusion phenotype previously described histologically [16,21], but its modest strength does not by itself establish a systemic–local causal axis. Taken together, these considerations indicate that the present findings are directionally consistent with prior work while differing in setting, endpoint, effect size, and the relative weight of PLR and that the apparent gain in discrimination requires external validation before it can be regarded as a true difference from the published literature rather than a property of this particular sample.

### 4.5. Study Limitations

This study has several limitations that should temper interpretation of the findings. First, the design is single-center, observational, and retrospective in nature, and the cohort size—although adequate for the principal univariate and multivariate analyses—remains modest at 87 patients, with sentinel lymph node biopsy data available in only 54 cases and only 21 recurrence events recorded over the follow-up window. The number of events constrains the multivariable Cox model to a maximum of two-to-three covariates per outcome under the conservative ten-events-per-variable rule [13], and although the principal three-variable model satisfied this constraint, exploratory models including additional covariates should be regarded as hypothesis-generating. Second, NLR and PLR are sensitive to acute conditions unrelated to the underlying tumor—including infection, recent surgery, smoking intensity, and use of corticosteroids or NSAIDs—and although eligibility criteria excluded patients with documented acute infection, residual confounding by subclinical inflammatory states cannot be fully excluded. Third, the baseline imbalance in BMI, hypertension, and diabetes mellitus across NLR strata, although not formally adjusted for in the multivariable models, raises the possibility of residual confounding by metabolic and cardiovascular comorbidity. Fourth, the median follow-up of 38.4 months is sufficient for analysis of recurrence-free survival but insufficient for robust estimation of overall survival, and the NLR threshold of 3.0—pre-specified from prior literature [7,9]—may not represent the optimal cut-off in other populations or melanoma subtypes. Prospective validation in an independent, ideally multi-center, cohort is required before NLR-based risk stratification is integrated into routine preoperative decision-making [30]. In addition, individual genetic predisposition—for example, germline variants such as CDKN2A or MC1R and constitutional differences in inflammatory and immune set-point—was not captured in this study and could influence both peripheral inflammatory ratios and melanoma behavior, representing a further potential confounder that future work should address. A specific concern is the risk of overfitting of the predictive models given the small sample size, and in particular the multivariable logistic regression, backward selection, and reclassification metrics that rest on only 54 biopsied patients with 13 positive nodes; with so few events per candidate variable, these models are unstable, likely optimistic, and were not internally or externally validated, so the reported AUCs, odds ratios, and reclassification statistics should be read as exploratory estimates rather than validated performance. A further important limitation is the absence of an independent validation cohort: all cut-offs, ROC estimates, regression models, and reclassification metrics were derived and tested within the same small single-center sample, so the results are internal and may be optimistic. External validation in separate cohorts is therefore essential before any of these estimates can be considered reliable.

### 4.6. Strengths and Limitations of the Study

The main strengths of this study are the use of routinely available, inexpensive biomarkers that require no specialized assay; the pre-specification of the primary analyses and subgroup comparisons; the coherent, biologically plausible gradient linking NLR to histopathologic severity, sentinel-node positivity, and recurrence; the consistent direction of effect across urban and rural strata; and the transparent reporting of both a literature-based and a data-derived NLR cut-off. The complete-case dataset for the core variables and the use of established prognostic-modelling and reclassification methods add further rigor to the descriptive findings.

These strengths are balanced by important limitations. The study is single-center, retrospective, and observational, with a modest cohort of 87 patients, only 54 sentinel-node biopsies, 13 positive nodes, and 21 recurrence events, and a median follow-up (38.4 months) that is adequate for recurrence-free but not overall survival. The small number of events limits the multivariable models and creates a real risk of overfitting and optimistic performance estimates. All cut-offs, ROC curves, regression models, and reclassification metrics were derived and tested within the same cohort, with no independent validation set. Selection is indication-driven for the SLNB analyses; the residence subgroups are underpowered, NLR and PLR are sensitive to sub-clinical inflammation, and unmeasured factors—including metabolic comorbidity and individual genetic predisposition—may confound the associations. The findings should therefore be regarded as exploratory and hypothesis-generating.

### 4.7. Future Directions

Several questions follow directly from these results and define an agenda for future work. First, do the NLR and NLR/PLR thresholds identified here retain their prognostic value when applied prospectively in independent, multi-center cohorts, and what is the optimal, generalizable cut-off? Second, can the incremental value of inflammatory ratios over Breslow thickness and AJCC stage be confirmed in adequately powered samples with formal internal and external validation, and does it translate into better-calibrated nomograms for sentinel-node positivity? Third, does pre-treatment inflammatory status add information to, or interact with, molecular and genetic markers (for example, BRAF status, tumor mutational burden, and germline predisposition) and with tumor-infiltrating lymphocyte grade? Fourth, is a high inflammatory ratio associated with response to adjuvant immunotherapy and with overall survival over longer follow-up? Finally, prospective studies should test whether inflammatory biomarkers can safely refine—rather than replace—sentinel lymph node biopsy decisions in borderline candidates. Until such validation exists, NLR and PLR should be viewed as promising investigational tools rather than as ready for routine clinical use.

## 5. Conclusions

In this single-center cohort of primary cutaneous melanoma, pre-treatment NLR ≥ 3.0 emerged as a robust and independent prognostic biomarker across the full clinico-pathologic continuum of the disease. High-NLR patients presented with markedly thicker tumors, higher mitotic rates, more ulceration, more frequent lymphovascular invasion, more sentinel-node positivity, and approximately four-fold higher recurrence rates over the 38.4-month median follow-up, which remained significant after adjustment for Breslow thickness and AJCC stage. A weighted composite of NLR and PLR provided incremental discrimination beyond Breslow thickness alone for preoperative prediction of sentinel-node positivity. These findings, derived from inexpensive, universally available components of the complete blood count, are best regarded as exploratory and hypothesis-generating: they suggest that NLR-based stratification could, in principle, complement preoperative risk assessment for primary cutaneous melanoma, but this potential clinical utility remains unproven. Given the small, single-center, retrospective design and the lack of external validation, the findings warrant confirmation before any clinical application and motivate prospective, multi-center validation in larger cohorts before formal integration into clinical decision pathways.

## Figures and Tables

**Table 1:** Baseline demographic and clinical characteristics by NLR group.

Variable	Low NLR (n = 47)	High NLR (n = 40)	*p*-Value	Test
Age, years (mean ± SD)	56.2 ± 13.7	61.4 ± 12.8	0.072	Welch’s *t*-test
Male sex, n (%)	26 (55.3)	24 (60.0)	0.831	Fisher’s exact
Rural residence, n (%)	22 (46.8)	21 (52.5)	0.751	Fisher’s exact
BMI, kg/m^2^ (mean ± SD)	26.1 ± 3.7	28.4 ± 4.2	0.008	Welch’s *t*-test
Current smoker, n (%)	9 (19.1)	14 (35.0)	0.140	Fisher’s exact
Hypertension, n (%)	18 (38.3)	23 (57.5)	0.106	Fisher’s exact
Type 2 diabetes, n (%)	6 (12.8)	11 (27.5)	0.119	Fisher’s exact
Trunk lesion, n (%)	16 (34.0)	19 (47.5)	0.469	Chi-square
Upper limb lesion, n (%)	11 (23.4)	8 (20.0)		
Lower limb lesion, n (%)	12 (25.5)	7 (17.5)		
Head/neck lesion, n (%)	8 (17.0)	6 (15.0)		

Values are reported as mean ± standard deviation or n (%). Overall lesion-site distribution was compared using the chi-square test.

**Table 2:** Histopathologic severity and AJCC stage distribution at definitive excision by NLR group.

Variable	Low NLR (n = 47)	High NLR (n = 40)	*p*-Value	Test
Breslow thickness, mm [IQR]	0.8 [0.4–1.3]	2.1 [1.2–3.4]	<0.001	Mann–Whitney U
Mitotic rate, /mm^2^ [IQR]	1.3 [0.7–1.9]	3.2 [1.8–4.7]	<0.001	Mann–Whitney U
Ulceration, n (%)	6 (12.8)	17 (42.5)	0.003	Fisher’s exact
Lymphovascular invasion, n (%)	2 (4.3)	9 (22.5)	0.014	Fisher’s exact
TILs absent, n (%)	8 (17.0)	19 (47.5)	0.004	Fisher’s exact
Stage 0, n (%)	4 (8.5)	1 (2.5)	<0.001	Chi-square
Stage I, n (%)	28 (59.6)	10 (25.0)		
Stage II, n (%)	11 (23.4)	14 (35.0)		
Stage III, n (%)	4 (8.5)	12 (30.0)		
Stage IV, n (%)	0 (0.0)	3 (7.5)		
Early stage (0/I), n (%)	32 (68.1)	11 (27.5)	<0.001	Fisher’s exact
Breslow > 2.0 mm, n (%)	7 (14.9)	23 (57.5)	<0.001	Fisher’s exact

Continuous variables are shown as median [IQR]. Stage distribution was compared using the chi-square test. TILs = tumor-infiltrating lymphocytes.

**Table 3:** Sentinel lymph node biopsy outcomes and consequent management by NLR group.

Variable	Low NLR (n = 47)	High NLR (n = 40)	*p*-Value	Test
SLNB performed, n (%)	23 (48.9)	31 (77.5)	0.009	Fisher’s exact
SLN positive, n/N (%) of biopsied	2/23 (8.7)	11/31 (35.5)	0.027	Fisher’s exact
Macrometastases (≥2 mm), n/N (%) of positive	0/2 (0.0)	5/11 (45.5)	0.487	Fisher’s exact
Number of positive nodes, median [IQR]	1.0 [1.0–1.0]	2.0 [1.0–3.0]	0.024	Mann–Whitney U
Extracapsular extension, n/N (%) of positive	0/2 (0.0)	3/11 (27.3)	1.000	Fisher’s exact
Final stage upgrade after SLNB, n (%)	2/23 (8.7)	11/31 (35.5)	0.027	Fisher’s exact
Adjuvant systemic therapy, n (%) of cohort	5 (10.6)	17 (42.5)	0.001	Fisher’s exact

Percentages for sentinel node positivity and downstream pathological characteristics are calculated among biopsied patients (denominators noted explicitly).

**Table 4:** Dose-response relationship between pre-treatment NLR quartile and pathological severity markers (n = 87).

Variable	Q1 (NLR < 1.8)	Q2 (1.8–2.6)	Q3 (2.7–3.8)	Q4 (>3.8)	*p* (Trend)
Patients, n	22	22	21	22	—
Breslow thickness, mm (median)	0.6	0.9	1.8	2.6	<0.001
Mitotic rate, /mm^2^ (median)	1.1	1.7	2.6	3.7	<0.001
Ulceration, n (%)	1 (4.5)	4 (18.2)	7 (33.3)	11 (50.0)	0.001
LVI, n (%)	0 (0.0)	2 (9.1)	3 (14.3)	6 (27.3)	0.013
TILs absent, n (%)	3 (13.6)	5 (22.7)	8 (38.1)	11 (50.0)	0.005
Advanced stage (III/IV), n (%)	0 (0.0)	4 (18.2)	5 (23.8)	10 (45.5)	<0.001
SLN positive/biopsied, n (%)	0/8 (0.0)	1/14 (7.1)	4/15 (26.7)	8/17 (47.1)	0.004
Serum LDH, U/L (median)	182.4	197.6	213.8	241.7	0.002

Trend *p*-values were computed using the Cuzick non-parametric test for trend (continuous variables) and the Cochran–Armitage test (proportions). LVI = lymphovascular invasion; TILs = tumor-infiltrating lymphocytes; SLN = sentinel lymph node; LDH = lactate dehydrogenase.

**Table 5:** Pathological severity, surgical findings, and recurrence by NLR group, stratified by urban versus rural residence.

	Urban (n = 44) Low NLR (n = 25)	Urban (n = 44) High NLR (n = 19)	*p*	Rural (n = 43) Low NLR (n = 22)	Rural (n = 43) High NLR (n = 21)	*p*
Breslow, mm (median [IQR])	0.7 [0.4–1.1]	2.0 [1.0–3.2]	<0.001	0.9 [0.5–1.4]	2.3 [1.4–3.6]	<0.001
Mitotic rate, /mm^2^ (median)	1.2	3.1	<0.001	1.4	3.4	<0.001
Ulceration, n (%)	3 (12.0)	7 (36.8)	0.066	3 (13.6)	10 (47.6)	0.027
Early stage (0/I), n (%)	18 (72.0)	6 (31.6)	0.018	14 (63.6)	5 (23.8)	0.013
Breslow > 2.0 mm, n (%)	3 (12.0)	10 (52.6)	0.004	4 (18.2)	13 (61.9)	0.005
SLN positive/biopsied, n	1/12	5/14	0.158	1/11	6/17	0.087
Recurrence at follow-up, n (%)	2 (8.0)	7 (36.8)	0.022	3 (13.6)	9 (42.9)	0.037

Between-NLR-group *p*-values within each residence stratum were computed using Mann–Whitney U (continuous variables) or Fisher’s exact test (categorical variables, due to small cell counts).

**Table 6:** Spearman rank correlations between systemic inflammatory ratios and continuous melanoma severity markers (n = 87).

Variable Pair	Spearman *ρ*	95% CI	*p*-Value	Strength *
NLR vs. Breslow thickness	0.487	0.307–0.638	<0.001	Moderate
NLR vs. mitotic rate	0.521	0.348–0.663	<0.001	Moderate
NLR vs. AJCC stage (ordinal)	0.461	0.276–0.617	<0.001	Moderate
NLR vs. TIL density (ordinal)	−0.298	−0.482 to −0.087	0.005	Weak
NLR vs. serum LDH	0.343	0.139–0.520	0.001	Weak
PLR vs. Breslow thickness	0.318	0.111–0.497	0.003	Weak
PLR vs. mitotic rate	0.297	0.088–0.481	0.005	Weak
PLR vs. AJCC stage (ordinal)	0.276	0.065–0.464	0.010	Weak
PLR vs. serum LDH	0.241	0.028–0.434	0.024	Weak
NLR–PLR composite vs. Breslow	0.524	0.352–0.666	<0.001	Moderate

* Magnitude interpretation: |ρ| < 0.30 weak; 0.30–0.50 moderate; >0.50 strong. The NLR–PLR composite was constructed as a weighted z-score combination of standardized NLR and PLR values.

**Table 7:** Discrimination, calibration, and reclassification metrics for prediction of sentinel lymph node positivity (n = 54 biopsied patients, 13 positives).

Predictor	AUC	95% CI	Cut-Off	Sens (%)	Spec (%)	*p* (DeLong) *
NLR (Youden)	0.773	0.638–0.908	1.98	100.0	46.3	ref
NLR (clinical, ≥3.0)	0.773	0.638–0.908	3.0	76.9	70.7	—
PLR (Youden)	0.707	0.564–0.850	182	46.2	92.7	0.413
PLR (clinical, ≥150)	0.707	0.564–0.850	150	53.8	78.0	—
NLR + PLR composite	0.829	0.708–0.950	z = 0.42	61.5	90.2	0.108
Breslow thickness (mm)	0.702	0.553–0.851	0.67	100.0	36.6	0.412
Composite vs. Breslow (DeLong)	—	—	—	—	—	0.041
NRI (continuous, vs. Breslow)	0.428	0.094–0.762	—	—	—	0.012
IDI (vs. Breslow)	0.118	0.028–0.208	—	—	—	0.010

* DeLong *p*-values reference each predictor against NLR (rows 3–6) and the composite against Breslow (final shaded row). NRI = net reclassification index (continuous version); IDI = integrated discrimination improvement. Sens and Spec for binary cut-offs are computed against the SLNB-positive endpoint.

**Table 8:** Cox proportional-hazards regression for recurrence-free survival (n = 87, 21 events).

Variable	Univariate HR	95% CI	*p*	Adjusted HR	95% CI	*p*
NLR ≥ 3.0	4.32	1.60–11.67	0.004	2.87	1.04–7.92	0.042
PLR ≥ 150	2.86	1.11–7.38	0.029	1.74	0.71–4.27	0.227
Breslow > 2.0 mm	5.17	1.96–13.63	0.001	3.14	1.21–8.17	0.019
Ulceration	4.08	1.54–10.82	0.005	2.43	0.96–6.16	0.061
Stage III/IV (vs. 0–II)	6.92	2.65–18.04	<0.001	4.06	1.51–10.93	0.005
Mitotic rate ≥ 2/mm^2^	3.71	1.34–10.25	0.011	1.92	0.78–4.71	0.155
LVI present	4.91	1.75–13.76	0.002	2.78	0.98–7.86	0.054
TILs absent	3.24	1.27–8.27	0.014	—	—	—
Age > 60 y	1.83	0.72–4.64	0.205	—	—	—
Male sex	1.36	0.55–3.39	0.508	—	—	—

HR = hazard ratio. Multivariable model: NLR, PLR, Breslow, ulceration, stage, mitotic rate, LVI. Variables marked “—“ were not retained in the multivariable model. Schoenfeld global *p* = 0.643; Harrell C-index = 0.792 (95% CI 0.715–0.869); −2 log-likelihood = 156.3; AIC = 170.3.

**Table 9:** Multivariable logistic regression for sentinel lymph node positivity (n = 54).

Variable	Univariate OR	95% CI	*p*	Adjusted OR	95% CI	*p*
**NLR ≥ 3.0**	5.78	1.78–18.77	0.004	3.42	1.17–9.99	0.024
**PLR ≥ 150**	4.62	1.40–15.27	0.012	2.18	0.74–6.43	0.157
**Breslow > 2.0 mm**	7.50	2.20–25.61	0.001	4.27	1.43–12.74	0.009
**Ulceration**	6.17	1.66–22.96	0.007	2.86	0.95–8.61	0.062
**Mitotic rate ≥ 2/mm^2^**	4.36	1.25–15.21	0.021	—	—	—
**LVI present**	8.91	1.62–49.13	0.012	—	—	—
**TILs absent**	4.05	1.20–13.69	0.024	—	—	—
**Age > 60 y**	1.84	0.55–6.17	0.323	—	—	—
**Male sex**	0.92	0.28–3.05	0.892	—	—	—

OR = odds ratio. Final multivariable model: NLR, PLR, Breslow > 2.0 mm, ulceration. Variables marked “—“ were eliminated during backward selection. Nagelkerke R^2^ = 0.421; Hosmer–Lemeshow *p* = 0.624; overall classification accuracy = 81.5%.

## Data Availability

The data presented in this study are available on request from the corresponding authors. The data are not publicly available due to privacy and ethical restrictions.
